# Supplementary material for: Content-rich biological network constructed by mining PubMed abstracts
Source: BMC Bioinformatics. 2004 Oct 8;5:147. doi: 10.1186/1471-2105-5-147 (PMC528731; doi:10.1186/1471-2105-5-147)
Supplement: Additional File 5 — The original Chilibot query results of the term "long-term potentiation (LTP)" and 22 other terms, limiting the latest references analyzed to the years 1990, 1995, 2000, and 2004. [file 1471-2105-5-147-S5.bz2 › chilibotAdditionalFile5/ltp2004/html/CAMKII.html]

 


**CAMKII** (Input: CAMKII ) 

---


**Google Searches:** Entire Web  | EDU domain only  | PDF files only

.

**External Links:** OMIM |
LocusLink
| Swissprot
| GeneCards

  
**Maps of CAMKII**

Simple
Complete
graph in
radiant
tree
square
layout.

**New Hypothesis !**

**Synonyms** 

- calcium calmodulin dependent protein kinase ii   [PubMed]

- camkii   [PubMed]

**Synopsis**

- These results suggest that activation of **CaMKII** and the concomitant phosphorylation of synapsin I induce insulin exocytosis from pancreatic beta cells.  J Pharmacol Sci, 2003 
  [23]

- These results suggest that activation of **CaMKII**, PKA and PKC may be crucial for the induction and the early phase  J NeurophysiolJ Neurophysiol, 2003 
  [23]

- The results suggest that **CaMKII** is involved in LTP induction, while CaMKIV and MAPK are rather involved in LTP maintenance.  Nippon Yakurigaku Zasshi, 2002 
  [23]

- These results suggest that estrogen action in the hippocampus is linked to **CaMKII** activation.  Brain Res, 2002 
  [23]

- we found that high concentrations of D 890 can directly inhibit calmodulin protein kinase II **CaMKII**, an enzyme required for LTP induction.  Hippocampus, 2002 
  [19]

- We show that Ras relays the NMDA R and **CaMKII** signaling that drives synaptic delivery of AMPA Rs during long term potentiation  [LTP] .  Cell, 2002 
  [19]

- Recent evidence shows that calcium calmodulin dependent kinase II **CaMKII** is upregulated early in the process of central sensitization.  Brain Res Brain Res Rev, 2002 
  [19]

- The data suggest that the dentate gyrus of chronically stressed rats may have developed a compensatory mechanism whereby calcineurin levels are reduced to maintain normal P **CaMKII** levels, which may be responsible for the normal early LTP of the dentate gyrus of chronically stressed rats.The results of this work will increase understanding of why certain brain regions are more resistant to deleterious effects of conditions that deteriorate learning and memory.  Neuroscience, 2003 
  [17]

- We conclude that the NR1 C0 region is a key site for recruiting **CaMKII** to the postsynaptic site, where it may act in concert with calmodulin to modulate the stimulatory role of alpha actinin interaction with the NMDA receptor.  J Biol Chem, 2002 
  [16]

- We describe how gene knock out technology was initially exploited to disrupt the calcium calmodulin dependent protein kinase IIalpha CaMKIIalpha gene and how refined knock in techniques later allowed an analysis of the role of distinct phosphorylation sites in **CaMKII**.  Philos Trans R Soc Lond B Biol Sci, 2003 
  [15]

- Together, these results support the interpretation that **CaMKII** contributes to the control of activity dependent structural plasticity.  J Neurosci, 2003 
  [14]

- The switch like response of a **CaMKII** PP1 system suggests that **CaMKII** and PP1 may function together as a simple molecular device that specifically translates only strong calcium signals into all or none potentiation of individual hippocampal synapses.  Proc Natl Acad Sci U S A, 2003 
  [13]

- These results demonstrate that in the hippocampus, **CaMKII** activation facilitates the induction of low frequency LTP, but at high levels of expression, compensatory mechanisms act to inhibit the induction of this form of LTP.  J Neurosci, 2002 
  [11]

- The results support a model in which locally stored **camkII** messenger ribonucleic acid is rapidly transported to dendritic spines and translated during long term potentiation  [LTP]  in behaving rats.  Eur J Neurosci, 2003 
  [11]

- In addition, it involves protein kinases, including both presynaptic and postsynaptic **CamKII** and probably MAP kinase.  J Neurosci, 2003 
  [10]
